# Simultaneous Maxillofacial, Abdominal, and Bone Marrow Involvement in a Pediatric Patient With Epstein-Barr Virus-Associated Burkitt Lymphoma: A Case From India

**DOI:** 10.7759/cureus.90010

**Published:** 2025-08-13

**Authors:** Vijayalakshmi Minumanuri, Paroma Agarwal, Navankur Chakma, Payal Yadav, Deepak Kumar, K Rajeshwari

**Affiliations:** 1 Pediatrics, Maulana Azad Medical College, Delhi, IND

**Keywords:** burkitt lymphoma, epstein-barr virus, fine needle aspiration cytology (fnac), india, non-hodgkin lymphoma

## Abstract

Burkitt lymphoma is a rare and aggressive B-cell origin non-Hodgkin lymphoma, which has been classified as a poorly differentiated lymphocytic lymphoma. Three major subtypes of Burkitt lymphoma have been identified, namely endemic, sporadic, and immunodeficiency-associated. While an abdominal (ileocecal) mass is the most common presentation of Burkitt lymphoma in sporadic populations, including the Indian subcontinent, involvement of the face/jaw/sinuses/ovaries is considered to be extremely rare.

We present the case of a three-year-old female child who presented with progressive, painless, bilateral cheek swelling with facial and periorbital puffiness and abdominal distension, which was associated with a high-grade fever and significant weight loss for 20 days. The simultaneous involvement of solid organs of the abdomen and face is a very rare presentation in the Indian population. Diagnosis was confirmed by fine needle aspiration cytology (FNAC) of cervical lymph nodes and immunohistochemistry (IHC) of bone marrow biopsy. The patient was diagnosed with Stage 4 Burkitt lymphoma.

Extra-abdominal involvement at presentation, particularly of the facial bones or sinuses, is uncommon in the Indian setting. This highlights the variety of Burkitt clinical presentations in India and the significance of early diagnosis.

## Introduction

Non-Hodgkin lymphomas are a heterogeneous group of lymphoproliferative malignancies originating from either B or T lymphocytes at various stages of maturation.

Burkitt lymphoma is a highly aggressive and uncommon B-cell non‑Hodgkin lymphoma that is categorized by the World Health Organization into three clinical variants: endemic, sporadic, and immunodeficiency‑associated. The endemic variant is seen in Equatorial Africa and usually presents as a rapidly growing jaw mass [1]. Sporadic variant is seen in the rest of the world, including India, and typically manifests as a large abdominal mass, most frequently involving the ileocecal region. Immunodeficiency-associated Burkitt lymphoma is seen in HIV-positive individuals, with predominant lymph nodal involvement [1].

Extra-abdominal involvement, particularly of the facial bones or paranasal sinuses, is seen in only 15% of cases of the non-endemic variant [2]. In India, Burkitt lymphoma has been reported to involve the jaw and abdomen in nearly equal proportions [3]. Although both sites are commonly affected individually, their simultaneous involvement at the initial presentation is exceedingly rare and, to the best of our knowledge, has not been well documented in existing literature. We describe a rare and aggressive case of Epstein-Barr virus (EBV)-positive sporadic Burkitt lymphoma in a young girl with simultaneous involvement of facial bones, solid organs of the abdomen, and bone marrow, emphasizing the diversity of clinical manifestations in the Indian setting and the importance of prompt diagnosis.

## Case presentation

History

A three-year-old female child, born out of a third-degree consanguineous marriage and third in birth order, presented to a tertiary care hospital. She had an uneventful birth history and was developmentally normal. Her chief complaints were progressive, painless, and bilateral cheek swelling with facial and periorbital puffiness for 20 days, associated with new-onset mouth breathing and snoring; progressive, painless, and generalized abdominal distension for 20 days; significant weight loss, easy fatigability, and progressive pallor for the same duration; and high-grade, intermittent fever spikes for one week.

Examination

**Figure 1:**
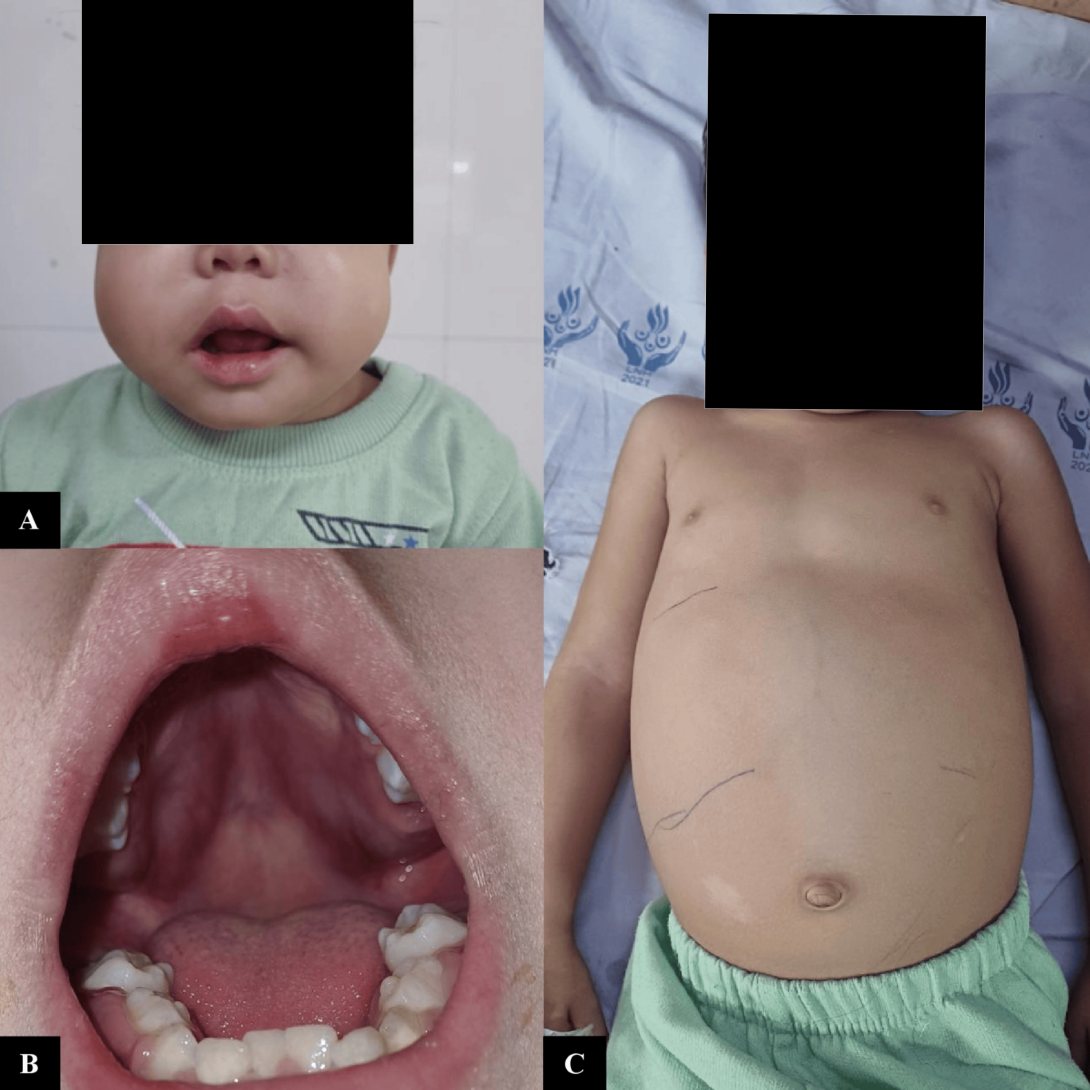
Examination A: bilateral nontender firm cheek swelling with facial and periorbital puffiness with depressed nasal bridge, anteverted nares, and mouth breathing; B: gum hypertrophy, with bilateral downwards protruding palate, with overcrowded teeth; C: gross abdominal distension with hepatosplenomegaly.

Physical findings showed pallor, with frontal bossing, a depressed nasal bridge, anteverted nares, and facial and periorbital puffiness. There was bilateral nontender, nonpitting, firm cheek swelling with tense, shiny overlying skin and prominent dilated veins over the swelling (Figure 1A). The mouth was frequently kept open, with snoring while sleeping. The oral cavity revealed overcrowded teeth, gum hypertrophy, and a downward protruding palate bilaterally, with normal tonsils, tonsillar pillars, and posterior pharyngeal wall (Figure 1B). No oral mucosal bleed was noted. Bilateral firm, nontender, mobile, non-matted, sub-centimetric lymph nodes were palpable in level 1b (submandibular), 2a and 2b (upper and mid jugular); the largest measuring 1×1.5 cm. There was no axillary or inguinal lymphadenopathy. Abdominal examination revealed a grossly distended abdomen with hepatosplenomegaly (liver-5 cm below the costal margin with a span of 10 cm and spleen-3 cm in the spino-umbilical line) (Figure 1C). The remaining systemic examination was unremarkable.

Investigations

**Figure 2:**
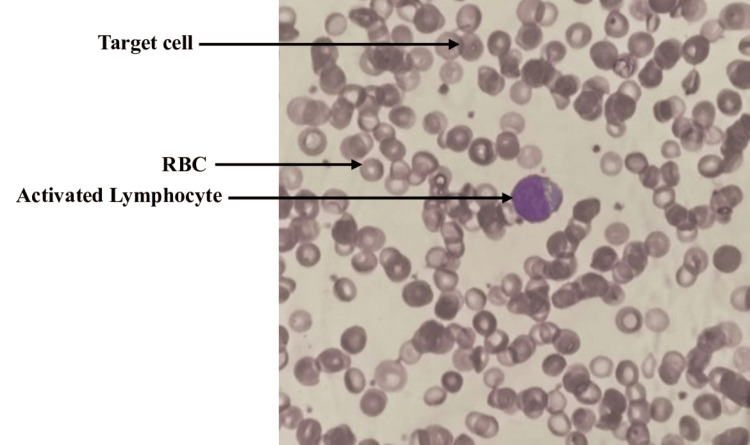
Peripheral smear shows activated lymphocytes in a background of normocytic-normochromic red blood cells with anisopoikilocytosis, with elliptical and target cells

Peripheral blood smear showed normocytic normochromic anemia and lymphocytic predominant WBCs with many activated lymphocytes (Figure 2).

Lactate dehydrogenase (LDH) and uric acid were elevated (Table 1). HIV serology of the child was nonreactive, and EBV serology was positive (IgM negative, IgG positive with high avidity). Laboratory findings have been listed in Table 1.

**Table 1:** Results of laboratory investigations HB: hemoglobin; HCT: hematocrit; DLC: differential leucocyte count; SGOT (AST): serum glutamic-oxaloacetic transaminase (aspartate aminotransferase); SGPT (ALT): serum glutamic-pyruvic transaminase (alanine aminotransferase); ALP: alkaline phosphatase; LDH: lactate dehydrogenase

Test	Result	Unit	Reference value (for age & gender)
Hematology			
HB	7.4	gm/dl	11-14
HCT	21	%	33-42
WBC	6600	cells/mm^3^	4000-11000
DLC			
Neutrophils	31	%	40-75
Lymphocytes	62	%	20-50
Monocytes	4	%	2-10
Eosinophils	3	%	1-6
Platelet	2.90 lakh	cells/mm^3^	1.5-4.0 lakh
Biochemistry			
Urea	21	mg/dl	9-22
Creatinine	0.3	mg/dl	0.2-0.43
Total serum bilirubin	0.2	mg/dl	0.05-0.4
SGOT (AST)	42	IU/L	21-44
SGPT (ALT)	16	IU/L	9-25
ALP	115	IU/L	156-369
LDH	2124	IU/L	192-321
Uric acid	12	mg/dl	1.8-4.9
Total protein	6.4	gm/dl	6.1-7.5
Serum albumin	3.6	gm/dl	3.8-4.7

Imaging

**Figure 3:**
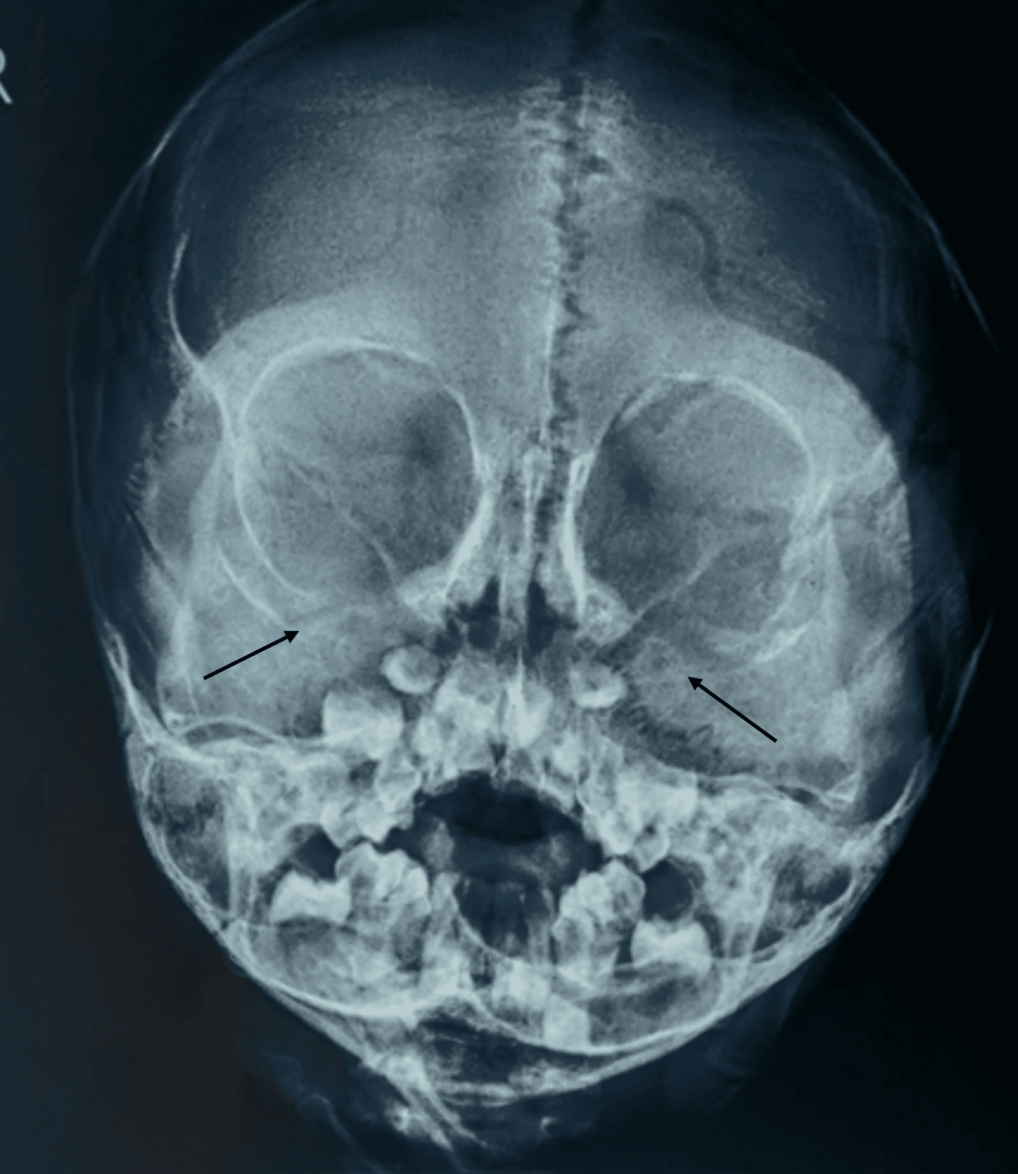
X-ray skull reveals homogenous enhancing masses in bilateral maxillary sinuses with extension into orbits (indicated by arrows)

**Figure 4:**
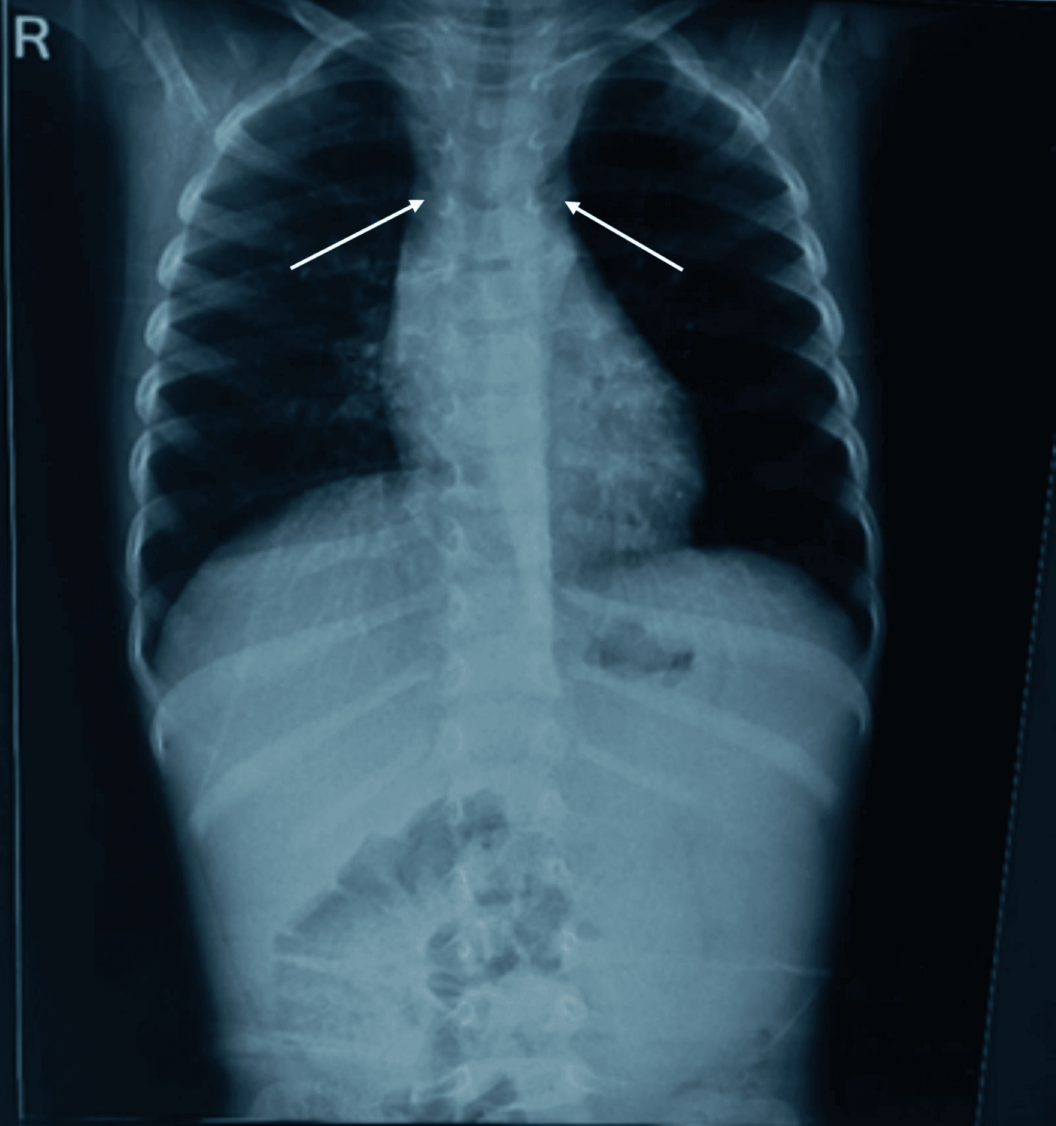
Chest X-ray reveals mediastinal lymphadenopathy with normal lung parenchyma (indicated by arrows)

X-ray of orbit and maxillary sinus showed a homogeneous soft tissue mass in bilateral maxillary sinuses with extension into orbits bilaterally (Figure 3). Chest X-ray revealed mediastinal lymphadenopathy (Figure 4).

USG of the abdomen and pelvis showed hepatosplenomegaly with well-defined solid lesions in bilateral adnexa measuring 2.3 cm × 1.7 cm on the right side and 2.2 cm × 1.9 cm on the left side with an echogenic focus seen in the periphery of the lesion, likely calcification, with significant internal vascularity. USG of the bilateral parotid glands showed bilateral parotid gland enlargement with multiple intraparotid lymph nodes.

Contrast-enhanced computed tomography (CECT) of the head, chest, abdomen, and pelvis showed enhancing soft tissue masses in bilateral maxillary sinuses and nasopharynx and bilateral orbits with extradural extension along the bilateral temporal region, mediastinal lymphadenopathy, solid lesions with smooth margins in bilateral adnexa, bulky bilateral kidneys, and retroperitoneal lymphadenopathy.

Histopathological and immunohistochemical findings

Bone marrow aspirate revealed aparticulate smears with near total (82-86%) replacement of marrow by atypical lymphoid cells, and biopsy revealed monomorphic large atypical cells replacing the marrow spaces with a high nucleocytoplasmic ratio, prominent nucleoli, and high mitotic activity.

**Figure 5:**
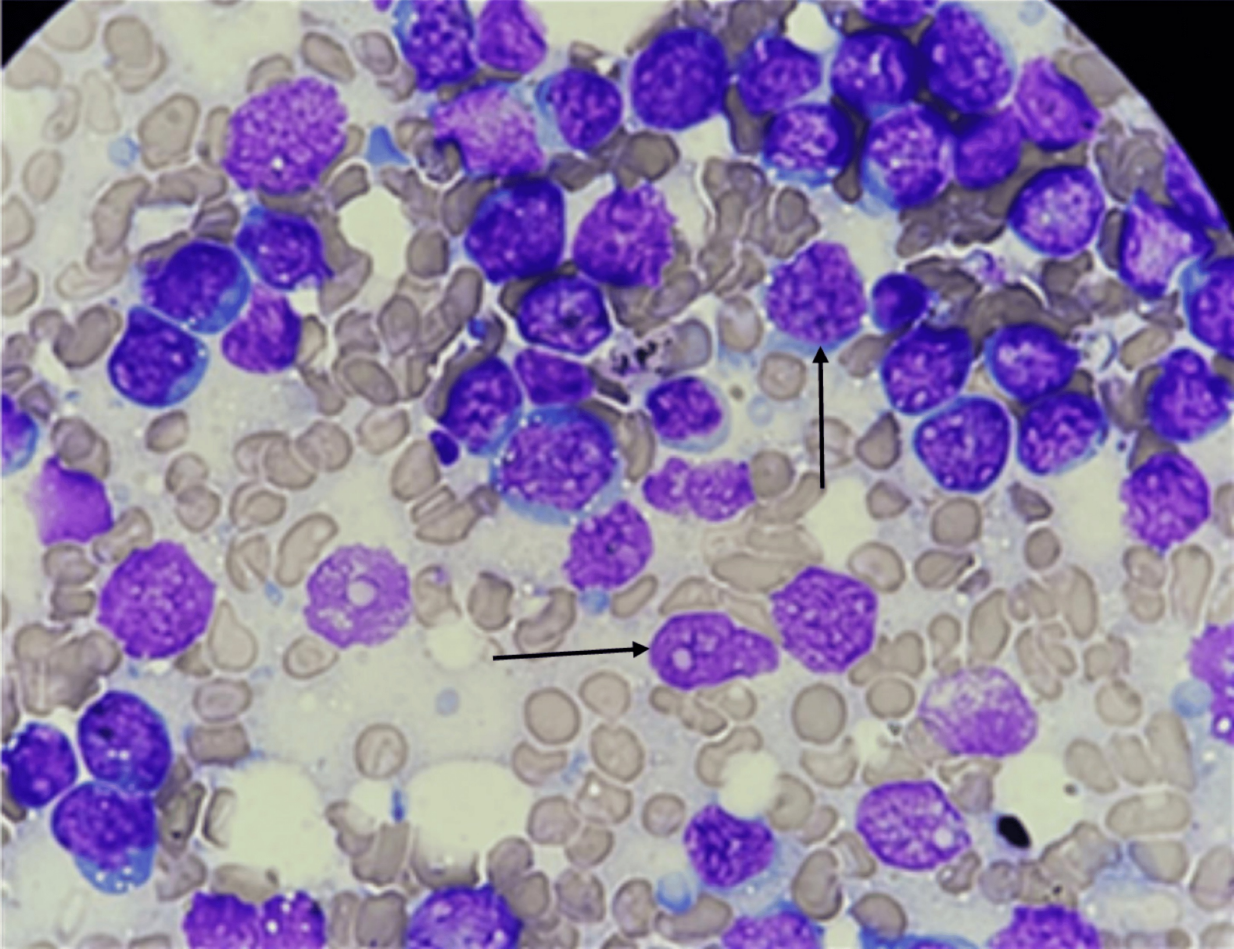
FNAC from cervical lymph node Atypical lymphocytes, 2.5 to three times the size of mature lymphocytes, with a high nuclear-cytoplasmic ratio, finely clumped chromatin, deep blue cytoplasm, and cytoplasmic non-coalescent vacuolation with one to two nucleoli with high mitotic activity (indicated by arrows). FNAC: fine needle aspiration cytology

Fine needle aspiration cytology (FNAC) from the submandibular lymph node revealed replacement of the lymph node by atypical lymphoid cells, the morphology of which was consistent with Burkitt lymphoma (Figure 5).

Immunohistochemistry (IHC) revealed positivity for CD38, CD20, BCL6, CD10, and CD19 with a Ki67 index of 100%, confirming the diagnosis of Burkitt lymphoma.

Based on the above findings, the child was diagnosed as a case of Burkitt lymphoma, Stage 4, as per Murphy and St Jude staging.

Treatment course

The child was started on IV hydration and allopurinol and was initiated on chemotherapy with the R-CODOX-M/IVAC regimen, but unfortunately, the child succumbed to the disease within two months of presentation.

## Discussion

Burkitt lymphoma is a highly aggressive monoclonal neoplasm of B‑lymphocytes, previously categorized as a poorly differentiated lymphocytic lymphoma. Sporadic variant accounts for 30-50% of childhood lymphomas and predominantly affects children and adolescents, with incidence peaks at 10 and 40 years. The male-to-female ratio is 3:1. The typical presentation of sporadic Burkitt lymphoma is a large abdominal mass, frequently involving the ileocecal region. Bone marrow infiltration is seen in 10-20% of cases, and EBV association is seen in 20-30% of cases [1].

Endemic Burkitt lymphoma has a peak incidence between three and eight years of age, and the male-to-female ratio is 2:1. The commonest site of disease presentation is the face, with multiple facial bone involvement, usually the maxilla and mandible [1]. Bone marrow infiltration is uncommon, and EBV association is seen in almost all the cases [1].

Burkitt lymphoma is rare in the Indian population. Occasional sporadic cases of Burkitt lymphoma have been reported across various regions of India. However, large related studies in children and young adults are very few. In an Indian study by Sarma S et al., out of 2505 cases of diagnosed lymphomas, 2.85% were Burkitt [4]. In a 10‑year study of 263 pediatric solid organ tumors in India by Pramanik et al., only two cases (0.76 %) were diagnosed as Burkitt lymphoma [5]. The clinical features of Burkitt lymphoma in Indian patients appear to fall between those of the sporadic and endemic variants [3,6,7]. Its association with EBV varies from 25% to 80% [5]. This case, too, could be classified as intermediate, exhibiting features of both endemic and sporadic forms, and had an EBV association.

The current case is a three-year-old female who presented with simultaneous involvement of the abdomen (atypically involving solid organs, ovaries, and kidneys) and face (maxillary sinus, nasopharynx, and orbit) with generalized lymphadenopathy (cervical, mediastinal, and retroperitoneal) and with infiltration of bone marrow at the time of presentation.

The final diagnosis of this patient was based on FNAC of the cervical lymph node, which revealed atypical lymphocytes with finely clumped chromatin, prominent nucleoli, and vacuolated cytoplasm, all of which are consistent with Burkitt lymphoma, and IHC, which revealed positive B cell markers and a Ki67 index of 100%. Several case reports of Burkitt lymphoma published from India were similarly diagnosed based on FNAC findings. Pathade SC et al., used FNAC of cervical lymph nodes to diagnose a 14-year-old with Burkitt lymphoma involving lymph nodes, CNS, and solid organs of the abdomen (liver, bilateral kidneys, and pancreas) [7]. Rodge H et al. also used FNAC of cervical lymph nodes to diagnose Burkitt in a 12-year-old with polyserositis, pancytopenia, and cervical lymphadenopathy [8]. However, despite such extensive involvement, neither case had facial involvement, which is unique in this case.

Diagnosis is primarily cytological, hinging on the classic “starry sky” appearance, alongside immunophenotyping that shows tumor cells positive for B‑cell markers (CD19, CD20, CD22, CD79a, CD38, PAX5) and germinal‑center markers (CD10, BCL6). Cells are negative for BCL2, CD5, TdT, and CD23. The Ki67 index, also called the mitotic index, is usually high (> 90%). The presence of vacuolated cytoplasm in the atypical lymphocytes with lipid vacuoles, which stain positive for adipophilin, also aids in diagnosis [9].

Burkitt is a rapidly proliferating tumor with a cell turnover time of less than 24 hours, making it a highly aggressive tumor with a high risk of spontaneous tumor lysis syndrome and acute renal failure even before initiation of chemotherapy, which was seen in this case too [10]. LDH and uric acid levels of the patient were markedly raised at presentation despite a short clinical history of only 20 days. The child at presentation had bone marrow infiltration and hence was diagnosed as Stage 4 lymphoma based on the St. Jude and Murphy staging system [11] and risk group C based on the FAB/LMB96 system [12].

The primary modality of treatment for Burkitt lymphoma is multi-agent systemic chemotherapy and/or immunotherapy, along with intrathecal chemotherapy. The popularly followed regimen is R-CODOX-M/IVAC, consisting of rituximab, cyclophosphamide, vincristine, doxorubicin, dexamethasone, cytarabine, and methotrexate, which was proven effective in the French American British Lymphoma Mature B cell 96 trial (FAB-LMB 96 trial) [12,13]. Patients are at high risk for tumor lysis syndrome and acute renal failure and hence require vigorous hydration, alkaline diuresis, electrolyte monitoring, and allopurinol (xanthine oxidase inhibitor) and/or rasburicase (recombinant urate oxidase) [10].

Burkitt lymphoma in children has a very good prognosis after initiation of chemotherapy, with a five-year survival rate of over 90%, which is higher compared to adults and elderly patients [12,13]. However, involvement of bone marrow or CNS or presence of baseline LDH levels greater than twice the upper limit of normal have been associated with poor prognosis and higher mortality, which were seen in this patient [14]. In this patient, there was 82-86% (>25%) infiltration of bone marrow, which was categorized in risk group C and defined as Burkitt leukemia [15]. In a study by Song JY et al., the presence of widespread Burkitt lymphoma with bone marrow involvement was found to have a five-year survival rate of only 18.4% [16]. The patient in the current case succumbed to the disease within two months of presentation, despite the initiation of chemotherapy, due to the advanced stage and widespread involvement of the tumor at presentation.

Recent trends in Burkitt lymphoma have shown a global rise in disease burden, calling for an urgent need for targeted interventions and health care strategies to diagnose and treat the disease [17].

## Conclusions

Burkitt lymphoma is a rare, highly aggressive B-cell NHL that often shows hybrid clinical features falling between sporadic and endemic forms. An early diagnosis is crucial for prompt and effective management. In developing countries such as India, where peripheral laboratories often lack advanced diagnostic tools, FNAC provides a safe, efficient, and affordable approach for the timely identification of Burkitt lymphoma. Prompt initiation of chemotherapy results in an excellent prognosis and a good five-year survival rate. However, the presence of bone marrow involvement and spontaneous tumor lysis syndrome (TLS) at presentation is associated with a poor prognosis. The rising burden of Burkitt lymphoma worldwide calls for an urgent need for targeted interventions and healthcare strategies to diagnose and treat the disease.
